# Electroconvulsive therapy in treatment resistant schizophrenia: Old beacon of hope when nothing else works

**DOI:** 10.1192/j.eurpsy.2022.2021

**Published:** 2022-09-01

**Authors:** C. Adão, A.A. Quintão, A. Velosa, P. Trindade, C. Almeida

**Affiliations:** 1 Centro Hospitalar de Lisboa Ocidental, Psychiatry Department, Lisboa, Portugal; 2 NOVA Medical School, Psychiatry And Mental Health, Lisboa, Portugal

**Keywords:** treatment resistant, schizophrénia, ECT

## Abstract

**Introduction:**

Electroconvulsive therapy (ECT) is one of the oldest psychiatric treatments used to this day. It is particularly useful in cases of schizophrenia resistant to treatment with antipsychotics. 49% of patients with schizophrenia experience little or no response with one trial of antipsychotics, 71% do not achieve remission and up to 20% of patients are also resistant to clozapine.

**Objectives:**

Description of a clinical case where ECT is used in the treatment of resistant schizophrenia and review of the literature.

**Methods:**

Description of a clinical case. Non systematic review of the literature, searching the terms “treatment resistant”; “schizophrenia”; “ect” in the databases Pubmed, Medline, Cochrane and Uptodate.

**Results:**

Male, 38-year-old patient, diagnosed with schizophrenia for 20 years, with history of multiple hospitalizations, institutionalized for 9 years. Treated with risperidone 50 mg intramuscular fortnightly, clozapine 750 mg daily, aripiprazol 30 mg daily and diazepam 10 mg daily. He presented with increased delusional intensity for a year. Hospitalized for treatment with ECT, submitted to 12 sessions with bitemporal stimuli, with effective convulsions. MoCA, PANSS and BPRS were applied before and after treatment, with an increase of 25% in MoCA and a decrease of 47.3% and 57.9% respectively, in the psychotic symptoms scales.

**Conclusions:**

We present a case of schizophrenia resistant to treatment with multiple antipsychotics, including clozapine. ECT was used, with clinically demonstrated efficacy. In the future, it might be interesting to study in detail the mechanism of action of this treatment with the goal of deepening the knowledge of the neurobiology of schizophrenia.

**Disclosure:**

No significant relationships.

